# 2840. Prevalence of Acute Uncomplicated Cystitis in Japan

**DOI:** 10.1093/ofid/ofad500.2450

**Published:** 2023-11-27

**Authors:** Meg Franklin, Maia R Emden, Elise Bauer, Naomi Sacks, Shinya Kawamatsu, Yoshiaki Kawano, Fanny S Mitrani-Gold, Ashish V Joshi, Shinyoung Ju, Madison T Preib

**Affiliations:** PRECISIONheor, Boston, MA, USA; Franklin Pharmaceutical Consulting, Cary, NC, USA, Boston, Massachusetts; PRECISIONheor, Boston, Massachusetts; PRECISIONheor, Boston, Massachusetts; PRECISIONheor, Boston, Massachusetts; GSK, Tokyo, Tokyo, Japan; GSK, Tokyo, Tokyo, Japan; GlaxoSmithKline plc., Collegeville, Pennsylvania; GlaxoSmithKline plc., Collegeville, Pennsylvania; GSK, Tokyo, Tokyo, Japan; GSK, Tokyo, Tokyo, Japan

## Abstract

**Background:**

Urinary tract infections (UTIs) are among the most common bacterial infections worldwide. Uncomplicated UTI, or acute uncomplicated cystitis (AUC), is an area of interest for antimicrobial stewardship in Japan due to high prevalence of community antibiotic use. This analysis estimated the annual prevalence of AUC in Japan from 2016 to 2021.

**Methods:**

This cross-sectional observational study used Japanese Medical Data Center (JMDC) data from January 1, 2016 to November 30, 2021 to examine the epidemiology of AUC in Japan. Female patients aged ≥18 to < 75 years with an AUC diagnosis claim (ICD-10-CM N30.0, N30.9, N39.0) in the outpatient setting, including emergency room, were included. The annual prevalence for each year of interest was calculated as the number of patients with ≥1 AUC diagnosis claim in the outpatient setting divided by the number of female patients in the JMDC claims database who were ≥18 years old; values were multiplied by 100,000 to obtain prevalence per 100,000 females. Prevalence was calculated for subgroups of patients aged ≥18–49 years and ≥50–74 years (age ranges chosen as a proxy for menopause status). The annual prevalence of AUC in the Japanese female population (≥18 years old) was projected for the years 2016–2021 using population data taken from the annual Japan census.

**Results:**

The mean annual prevalence of AUC among women in Japan 2016–2021 was 7421 cases per 100,000 females (Table 1). The prevalence of AUC was higher for women aged ≥50 years at index compared with those aged < 50 years, with annual rates of 8716 vs 6273 cases per 100,000, respectively (Table 1). Annual prevalence projections of AUC in Japan (2016–2021) ranged from 2.6–3.9 million women, with 6.0–8.6% of the female population affected each year (Figure). Among patients who experienced recurrent AUC (≥2 AUC episodes in 12 months), the mean number of episodes per year was 3.45 (index + 2.45 recurrent episodes; [Table 2]).

**Figure:**
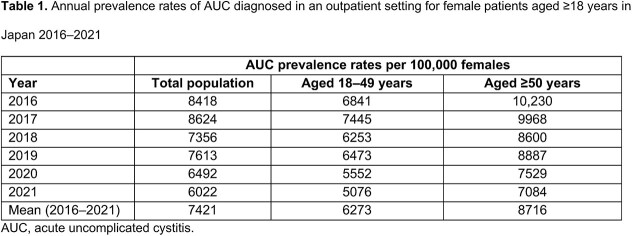

**Conclusion:**

Our study shows that the burden of AUC among female patients in Japan is moderate, with annual prevalence between 6.0 and 8.6%, and higher prevalence among women over the age of 50. Patients with AUC recurrence averaged 3.45 episodes per year.

**Figure:**
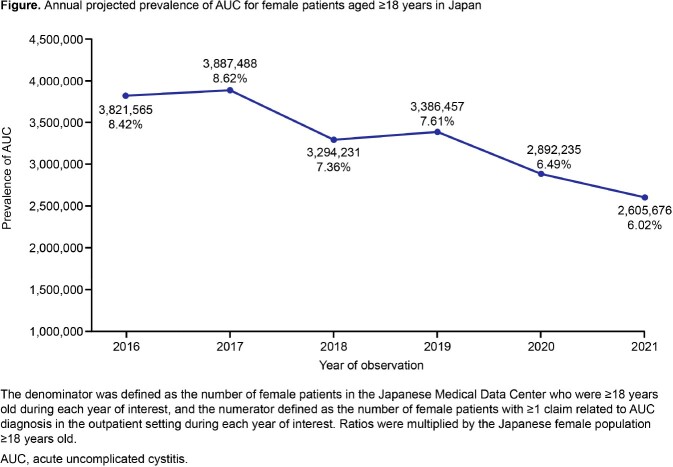

**Figure:**
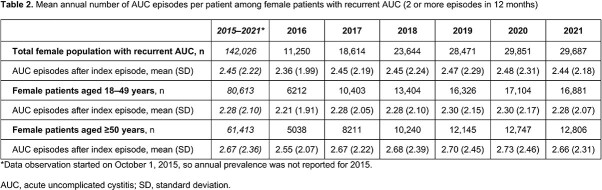

**Disclosures:**

**Meg Franklin, PharmaD, PhD**, Franklin Pharmaceutical Consulting: Owner and President|Franklin Pharmaceutical Consulting: Ownership Interest|GSK: Grant/Research Support|PRECISIONheor: Contractor **Maia R. Emden, BA**, GSK: Grant/Research Support|PRECISIONheor: Employee **Elise Bauer, MS**, GSK: Grant/Research Support|PRECISIONheor: Employee **Naomi Sacks, PhD**, GSK: Grant/Research Support|PRECISIONheor: Employee **Shinya Kawamatsu, PhD**, GSK: Employee|GSK: Stocks/Bonds **Yoshiaki Kawano, MD, PhD**, GSK: Employee|GSK: Stocks/Bonds **Fanny S. Mitrani-Gold, MPH**, GSK: Employee|GSK: Stocks/Bonds **Ashish V. Joshi, PhD**, GSK: Employee|GSK: Stocks/Bonds **Shinyoung Ju, MS**, GSK: Employee|GSK: Stocks/Bonds **Madison T. Preib, MPH**, GSK: Employee|GSK: Stocks/Bonds

